# The Potential Role of HMGB1 Release in Peritoneal Dialysis-Related Peritonitis

**DOI:** 10.1371/journal.pone.0054647

**Published:** 2013-01-24

**Authors:** Shirong Cao, Shu Li, Huiyang Li, Liping Xiong, Yi Zhou, Jinjin Fan, Xueqing Yu, Haiping Mao

**Affiliations:** 1 Department of Nephrology, The First Affiliated Hospital, Sun Yat-sen University, Key Laboratory of Nephrology, Ministry of Health, Guangzhou, China; 2 Department of Rheumatology, The Second Xiangya Hospital, Central South University, Changsha, Hunan, China; University of Leicester, United Kingdom

## Abstract

High mobility group box 1 (HMGB1), a DNA-binding nuclear protein, has been implicated as an endogenous danger signal in the pathogenesis of infection diseases. However, the potential role and source of HMGB1 in the peritoneal dialysis (PD) effluence of patients with peritonitis are unknown. First, to evaluate HMDB1 levels in peritoneal dialysis effluence (PDE), a total of 61 PD patients were enrolled in this study, including 42 patients with peritonitis and 19 without peritonitis. Demographic characteristics, symptoms, physical examination findings and laboratory parameters were recorded. HMGB1 levels in PDE were determined by Western blot and ELISA. The concentrations of TNF-α and IL-6 in PDE were quantified by ELISA. By animal model, inhibition of HMGB1 with glycyrrhizin was performed to determine the effects of HMGB1 in LPS-induced mice peritonitis. *In vitro*, a human peritoneal mesothelial cell line (HMrSV5) was stimulated with lipopolysaccharide (LPS), HMGB1 extracellular content in the culture media and intracellular distribution in various cellular fractions were analyzed by Western blot or immunofluorescence. The results showed that the levels of HMGB1 in PDE were higher in patients with peritonitis than those in controls, and gradually declined during the period of effective antibiotic treatments. Furthermore, the levels of HMGB1 in PDE were positively correlated with white blood cells (WBCs) count, TNF-α and IL-6 levels. However, pretreatment with glycyrrhizin attenuated LPS-induced acute peritoneal inflammation and dysfunction in mice. In cultured HMrSV5 cells, LPS actively induced HMGB1 nuclear-cytoplasmic translocation and release in a time and dose-dependent fashion. Moreover, cytosolic HMGB1 was located in lysosomes and secreted via a lysosome-mediated secretory pathway following LPS stimulation. Our study demonstrates that elevated HMGB1 levels in PDE during PD-related peritonitis, at least partially, from peritoneal mesothelial cells, which may be involved in the process of PD-related peritonitis and play a critical role in acute peritoneal dysfunction.

## Introduction

Peritoneal dialysis (PD) is the most important home dialysis therapy for patients with end-stage renal disease. Although the rate of peritoneal dialysis-related peritonitis has been significantly reduced, it remains a major complication of PD [1]. The repeated or severe peritonitis may cause peritoneal membrane dysfunction and eventually leads to dropout of PD.

The peritoneum is composed of an extensive monolayer of mesothelial cells resting upon a thin basement membrane. During peritonitis, mesothelial cells are activated by proinflammatory cytokines, such as tumor necrosis factor α (TNF-α) and interleukin-6 (IL-6) derived from peritoneal macrophages, and play critical roles in amplification of peritoneal inflammation though release of many proinflammatory cytokines and mediators [2].

High mobility group box protein 1 (HMGB1), a ubiquitous nonhistone nuclear protein, can be released actively by innate immune cells (macrophages, monocytes) or other cell types as well as passively by injured and necrotic cells [3], [4]. During systemic inflammation, TNF-α and IL-6 regulate HMGB1 release from various cells. Once released, HMGB1 may serve as a mediator of delayed endotoxin lethality and systemic inflammation, contributing to disease pathogenesis by upregulation of endothelial adhesion molecules, stimulation of epithelial cell barrier failure and enhancing the synthesis of proinflammatory cytokines [5], [6]. HMGB1 is not detectable in serum of normal subjects, but it is significantly increased in clinical inflammatory conditions such as sepsis, rheumatoid arthritis, and chronic kidney disease [7], [8], [9], [10]. Administration of HMGB1 via intracerebroventricular, intratracheal, intraperitoneal and intraarticular routes induces marked inflammatory responses, and activates various innate immune cells [6]. At the same time, targeting HMGB1 with either antibodies or specific antagonists has been demonstrated to blunt inflammatory response and confer protective effects in animal models, including lethal endotoxemia or sepsis, collagen-induced arthritis, and ischemia-reperfusion induced tissue injury [5], [11], [12], [13]. However, the potential role of HMGB1 in PD-related peritonitis has not been investigated.

In this study, we hypothesized that HMGB1 levels would be elevated in PDE and associated with peritonitis in PD patients. We also explored the effect of HMGB1 on peritoneal function in LPS-induced peritonitis in mice and further examined the source and the process of HMGB1 release using human peritoneal mesothelial cell line (HMrSV5) *in vitro*.

## Results

### Patient Characteristics

The baseline clinical characteristics of continuous ambulatory peritoneal dialysis (CAPD) patients with and without peritonitis were presented in Table 1. There were no significant differences in mean age, gender, the percentage of patients with diabetes, dialysis duration, hemoglobin (Hb), Kt/V_urea_, and residual glomerular filtration rate (GFR) between patients with or without peritonitis. However, patients with peritonitis had a higher high-sensitivity C-reactive protein (hsCRP) and lower levels of serum albumin (Alb) as compared with the control patients (*P*<0.05). The above results were similar in patients with Gram-positive or Gram-negative peritonitis.

**Table 1:** Patients Characteristics.

Characteristic	No peritonitis(n = 19)	Peritonitis(n = 42)	G^+^ peritonitis(n = 27)	G^−^ peritonitis(n = 15)
Age (years)	48.43±18.67	55.23±15.25	56.70±15.30	52.75±15.33
Gender (%, male)	57.9	61.9	66.7	53.3
Cause of ESRD (%)				
Diabetes mellitus	26.3	23.8	25.9	20.0
Glomerulonephritis	52.6	64.3	55.6	60
Other	21.1	11.9	11.1	20.0
PD duration(month)	22(6, 25)	11(4,17)	9(3,16)	12(9,24)
BMI (kg/m^2^)	22.51±2.31	22.71±2.72	23.43±2.22	21.51±3.11^b^
Hb (g/L)	100.75±23.93	95.4±21.25	93.21±23.31	99.10±22.12
Serum albumin (g/L)	35.67±3.57	31.55±5.26^a^	31.66±4.71^a^	31.35±6.24^a^
hs-CRP (mg/dL)	2.52(0.74,7.17)	9.28(3.88,12.21)^a^	8.00(4.07,11.82)	10.74(3.56,12.90*)* a
Total Kt/V	2.12(1.95,2.48)	2.20(1.97,2.73)	2.15(1.95,2.61)	2.23(2.20,2.73)
rGFR (ml/min/1.73 m^2^)	2.18(0.77,2.37)	2.06(1.43,3.47)	3.01(1.67,3.68)	2.1(0.96,2.69)

Note: ESRD, end-stage renal disease; PD, peritoneal dialysis; BMI, body mass index; Hb, haemoglobin; hsCRP, high-sensitivity C-reactive protein; Kt/V, solute clearance as a dialysis adequacy index; rGFR, residual glomerular filtration rate calculated by mean of creatinine and urea clearance.

Characteristics are presented as the mean ± SE or as median (interquartile range) for continuous variables and percentages for categorical variables.

a *p<0.05, vs* no peritonitis group.

b *p<0.05,vs* Gram-positive peritonitis group.

### Levels of HMGB1 in PDE during Peritonitis

**Figure 1:**
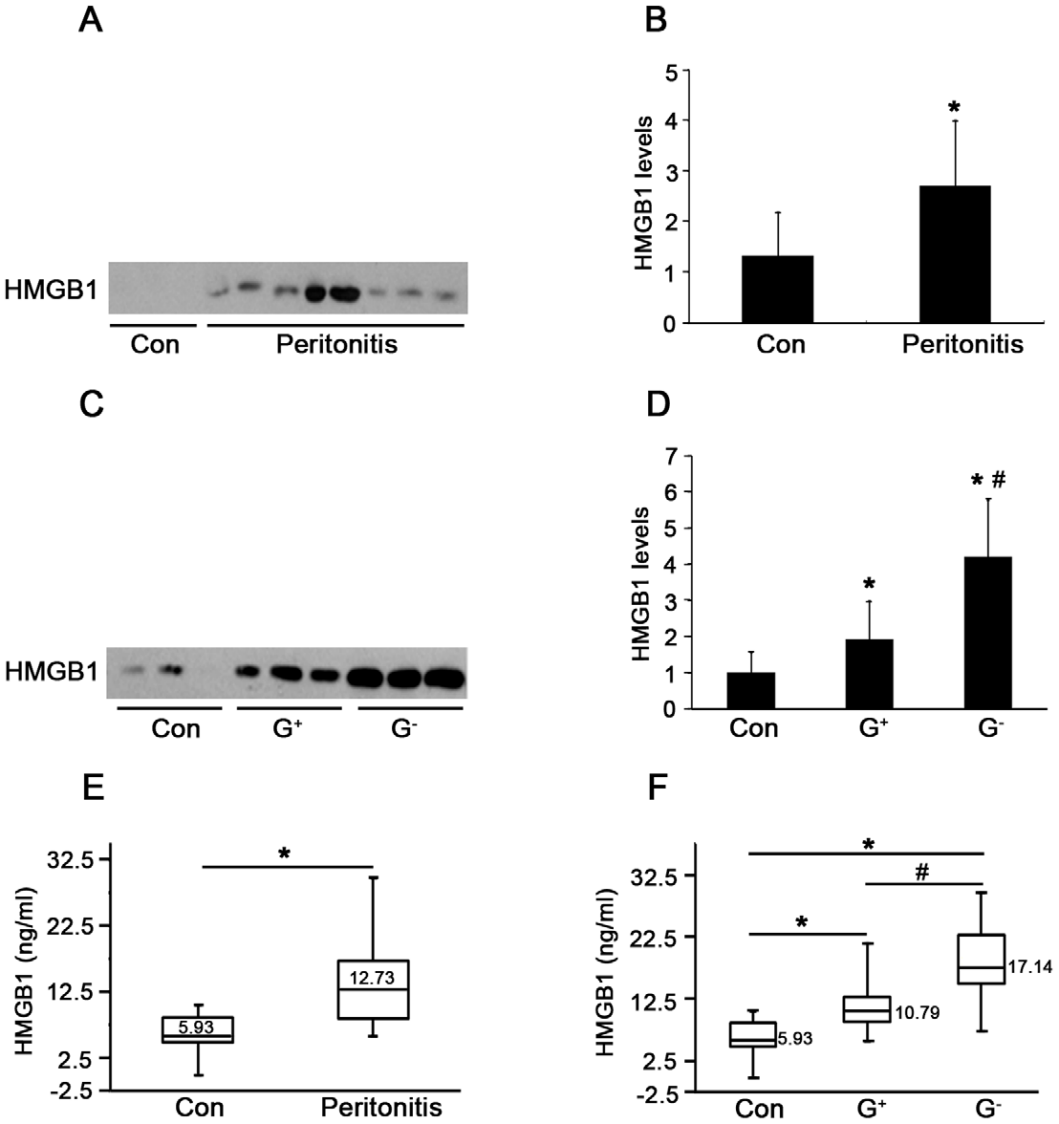
HMGB1 levels in peritoneal dialysis effluents (PDE). (A) Levels of HMGB1 in PDE of patients with or without peritonitis were detected by western blotting. PD patients without peritonitis served as controls (Con). (B) Densitometry of HMGB1 in immunoblots. Data are means ± SE (*n = *3), *P<0.05 *versus* control subjects. (C) Representative immunoblot for HMGB1 in PDE among patient subgroups, including patients without peritonitis, with Gram-positive (G^+^) and Gram-negative (G^−^) peritonitis. (D) Quantitative determination of the relative abundance of HMGB1 protein among different groups. Data are means ± SE (*n = *3), *P<0.05 *versus* control subjects. (E) Levels of HMGB1 in PDE of patients with or without peritonitis were quantified by ELISA. (F) Levels of HMGB1 in PDE among patient subgroups were assayed by ELISA. The box plot in E and F represents (from the top) values of the maximum, the third quartile, the median, the first quartile and the minimum, respectively (n = 4). *P<0.05 *versus* no peritonitis,^ #^ *P*<0.05 *versus* Gram-positive peritonitis.

**Figure 2:**
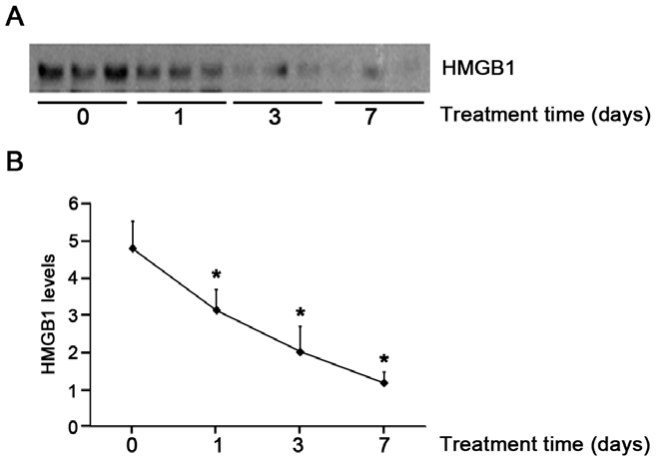
Serial changes in HMGB1 levels in PDE during peritonitis. (A) Representative HMGB1 immunoblot on PDE samples after antibiotic treatment. (B) Quantitative determination of relative HMGB1 levels in PDE after treatment. Data are expressed as mean ± SE from 3 independent experiments, *P<0.05 *versus* HMGB1 levels before treatment.

To determine whether HMGB1 levels are elevated in PD-related peritonitis, intraperitoneal HMGB1 concentrations were first determined by immunoblot analysis. As shown in Figure 1A and B, the levels of HMGB1 were significantly elevated in PDE samples of patients with peritonitis as compared with the controls. Moreover, levels of HMGB1 were significantly higher in patients with Gram-negative than those with Gram-positive peritonitis (Fig. 1C and D). HMGB1 levels in PDE samples were further confirmed by specific ELISA kits. Consistent with results obtained by immunoblot analysis, HMGB1 levels in PD patients with peritonitis were significantly increased compared to the controls (12.73 *versus* 5.93 ng/ml, *P*<0.01) (Fig. 1E), and also significantly higher in patients with Gram-negative peritonitis than those with Gram-positive peritonitis (17.14 ng/ml *versus* 10.79 ng/ml, *P*<0.01) (Fig. 1F). Importantly, dynamic observation of HMGB1 levels in peritonitis patients showed that high levels of HMGB1 were evident at the onset of peritonitis, gradually diminished and barely captured on day 7 after effective antibiotic treatment (Fig. 2A and B). Taken together, these findings suggest that increased PDE HMGB1 levels are associated with the severity of PD related-peritonitis, and LPS, endotoxin from Gram-negative bacteria may play additional direct role in stimulating HMGB1 secretion.

### Levels of TNF-α and IL-6 and their Correlation with HMGB1 in PDE

**Figure 3:**
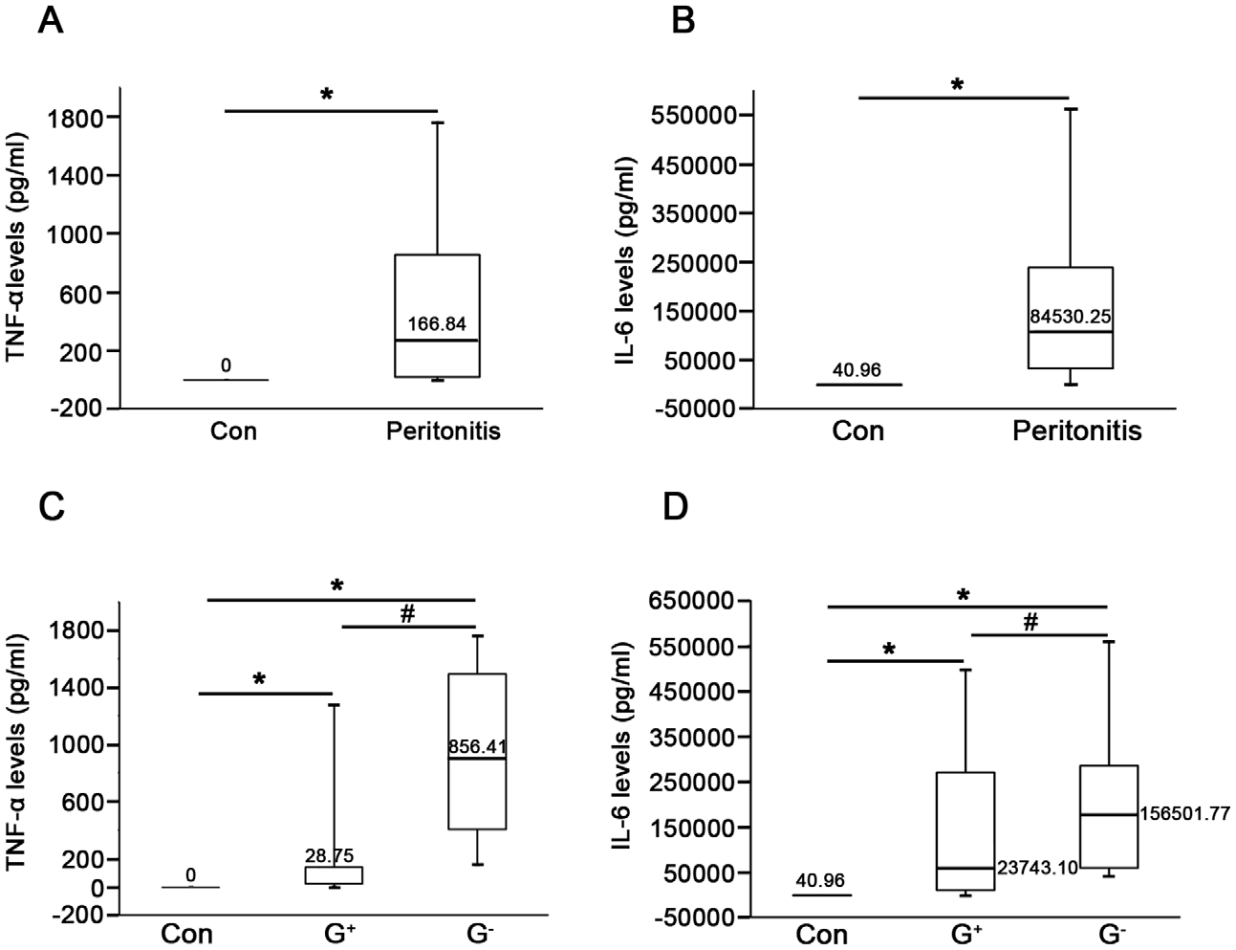
Levels of TNF-α and IL-6 in PDE during peritonitis. TNF-α levels (A) and IL-6 levels (B) in PDE were measured by ELISA from PD patients with or without peritonitis. The concentrations of TNF-α (C) and IL-6 (D) in PDE among subgroups of patients were determined by ELISA. The values represented the maximum, the third quartile, the median, the first quartile and the minimum, respectively (n = 4). *P<0.05 *versus* no peritonitis, ^#^ *P*<0.05 *versus* Gram-positive peritonitis.

**Figure 4:**
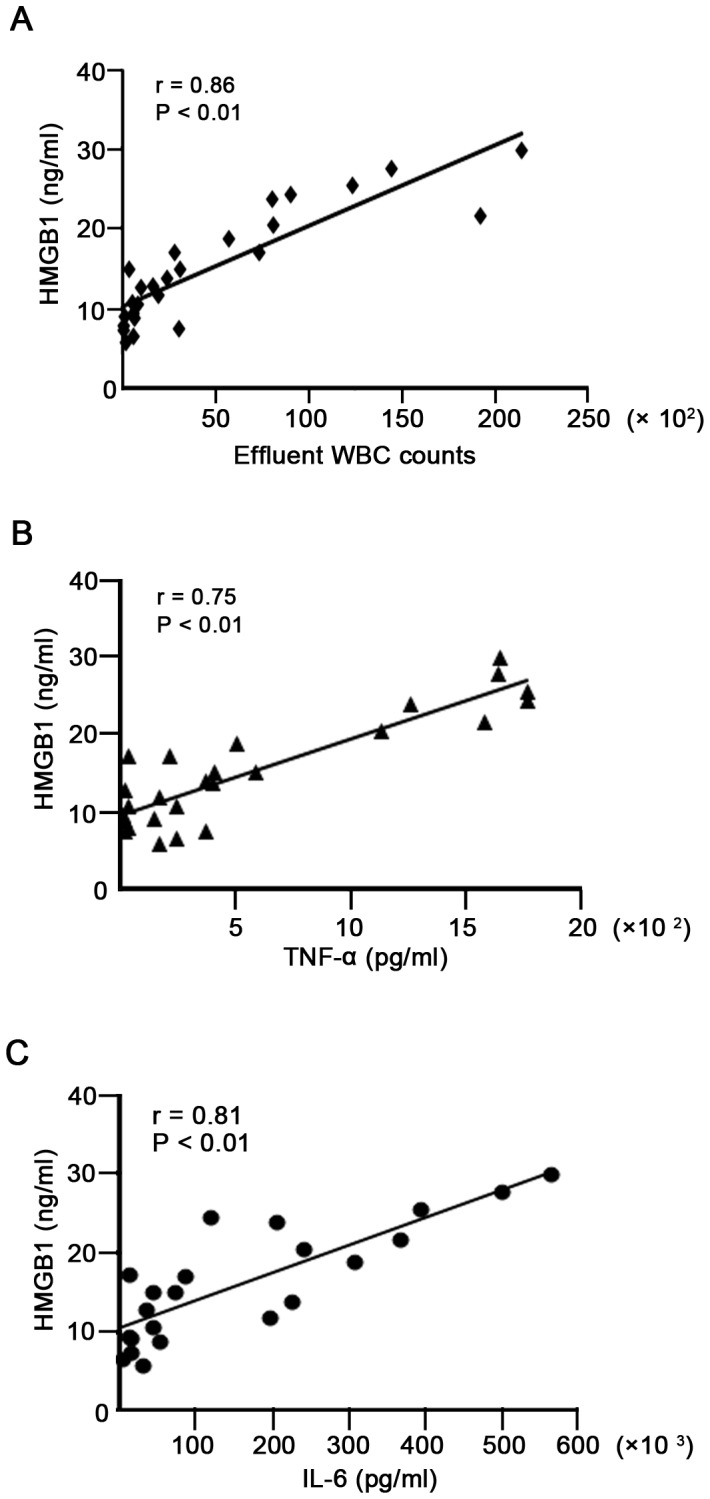
Correlation between PDE levels of HMGB1 and WBCs as well as cytokines during peritonitis. (A) Correlation between levels of HMGB1 and WBC counts in PDE (*r = *0.86, *P<*0.01). (B) Correlation between levels of HMGB1 and TNF-α in PDE (*r = *0.75, *P<*0.01). (C) Correlation between levels of HMGB1 and IL-6 in PDE (*r = *0.81, *P<*0.01).

In parallel analyses, we examined both TNF-α and IL-6 levels in PDE of the first day of peritonitis by ELISA. As shown in Figure 3A and B, levels of TNF-α and IL-6 in PDE of controls were almost undetectable, whereas levels of both cytokines markedly elevated in peritonitis patients. Similarly, PDE levels of TNF-α and IL-6 were higher in patients with Gram-negative as compared to Gram-positive peritonitis (*P*<0.01, Fig. 3C and D). Further, there was a significant positive correlation between HMGB1 levels and WBC counts (r = 0.86, *P*<0.01, Fig. 4A), TNF-α level (r = 0.75, *P*<0.01, Fig. 4B) as well as IL-6 level (r = 0.81, *P*<0.01, Fig. 4C) in PDE of patients with peritonitis.

### Inhibition of HMGB1 Expression Attenuated LPS-induced Peritoneal Dysfunction

**Figure 5:**
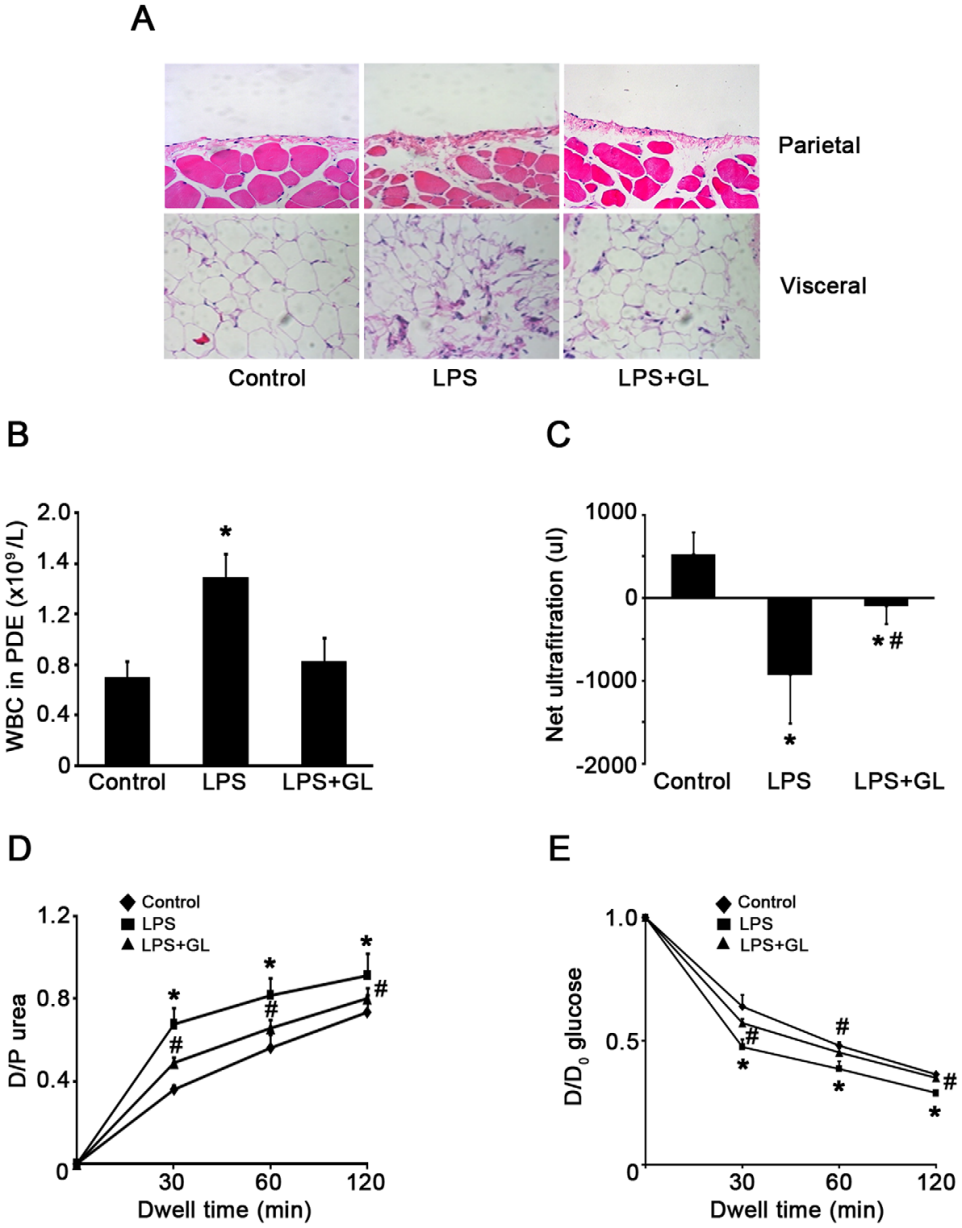
Effects of HMGB1 inhibitor on peritoneal inflammation and function. (A) Representative HE staining showed morphological changes and inflammatory infiltrate in both parietal and visceral peritoneum in each condition. Original magnification×200. (B) The total number of WBCs and the percentage of leukocytes in PDE among different group. (C–E) PD transport parameters to ultrafiltration, urea and glucose among different groups. Values are expressed as net ultrafiltration, D/P urea or D/D_0_ glucose. Data in C, D and E are mean ± SE (*n = *6), *P<0.05 *versus* control, # P<0.05 *versus* LPS-treated without glycyrrhizin (GL) administration.

Since elevated expression of HMGB1 in PDE was associated with the presence of PD-related peritonitis, we further evaluated whether HMGB1 could play a role in peritoneal function during peritonitis. To this end, acute peritonitis in mice was generated by intraperitoneal injection of LPS as previously described [14]. Compared with the control group, LPS administration significantly enhanced the peritoneal edema, recruitment of inflammatory cells in both the parietal and visceral peritoneum in mice (Fig. 5A). Further, the total number of WBC in dialysate was elevated (Fig. 5B) and the percentage of neutrophils increased (7.50±1.24% *versus* 25.00±5.85%, *P*<0.05) in parallel with a decrease in lymphocytes (72.70±7.90% *versus* 55.50±6.20%, *P*<0.05). Mice with acute LPS-associated peritonitis were accompanied by loss ultrafiltration (Fig. 5C), a progressive increase in the dialysate to plasma ratio for urea (Fig. 5D) and a progressive reabsorption of glucose from the dialysate (Fig. 5E). Pretreatment with glycyrrhizin, a known HMGB1 inhibitor [15], significantly attenuated these alterations, although glycyrrhizin did not affect LPS-induced increase in the percentage of neutrophils (data not shown). These findings suggest that inhibition of HMGB1 may provide protection against LPS-induced acute peritoneal inflammation and dysfunction. Of note, glycyrrhizin per se did not cause obvious cell toxicity in mice (data not shown).

### LPS Induced HMGB1 Release and Cytoplasmic Translocation in HMrSV5 Cells

**Figure 6:**
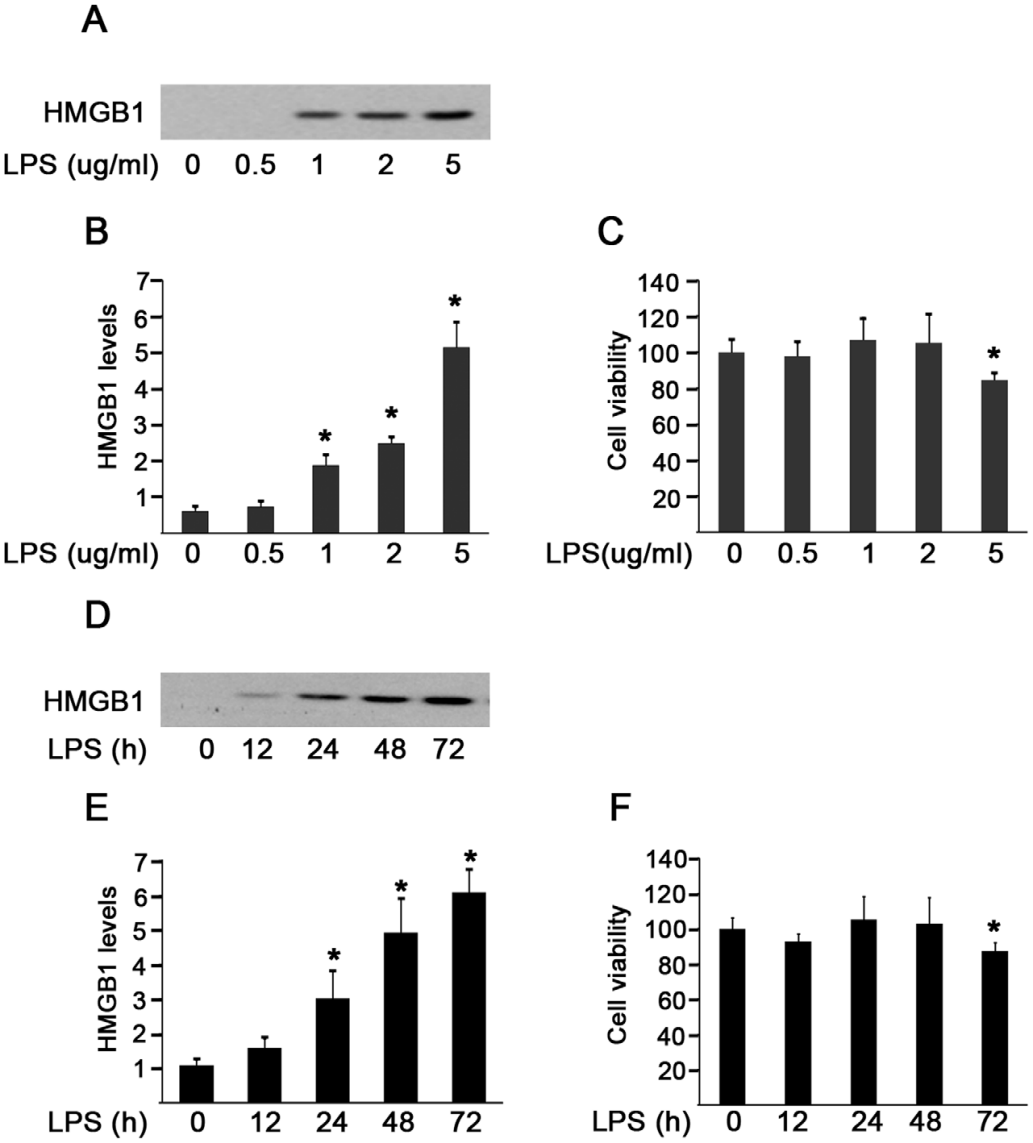
Effects of LPS on HMGB1 release in HMrSV5 cells. (A) Cells were treated with LPS at various concentrations for 48 hr. Cell culture media were collected and analyzed by immunoblotting with HMGB1 antibody. (B) Densitometry of HMGB1 proteins in immunoblots. (C) Cell viability was evaluated by MTT assay after treatment with LPS at the indicated concentrations for 48 hr. (D) Immunoblot analysis of HMGB1 in cell culture supernatants following 2 µg/ml LPS stimulation for the indicated time. (E) Quantitative determination of the relative abundance of HMGB1 proteins among different groups. (F) Cell viability was assayed by MTT at different time following LPS incubation. Data in B, C, E and F are expressed as mean ± SE (*n = *6). *P<0.05 *versus* control group.

Given that HMGB1 is released by a variety of activated immune and non-immune cells [16], [17], [18] and peritonitis can cause injury to mesothelial cells, it would be of interest to know whether the elevated HMGB1 in PDE of patients with peritonitis can be directly released from damaged peritoneal mesothelial cells. Because of the significantly higher release of HMGB1, TNF-α and IL-6 in Gram-negative peritonitis, LPS was used to examine HMGB1 release in peritoneal mesothelial cells. We found that LPS stimulation for 48 hr caused a dose-dependent active HMGB1 release in culture media from HMrSV5 cells (Fig. 6A and B). Notably, the release of HMGB1 was independent on cell death at the dose of LPS from 0.5 to 2 µg/ml, because it did not significantly affect cell viability (Fig. 6C). However, a high dosage of LPS (5 µg/ml) exhibited cytotoxicity and consequently triggered a more pronounced, robust HMGB1 release, possibly as a result of both active and passive HMGB1 release (Fig. 6A, B and C). In addition, exposure of cells to LPS (2 µg/ml) induced active HMGB1 release in a time-dependent fashion within 48 hr, since it showed a cytotoxic effect on cells at 72 hr after LPS treatment (Fig. 6D, E and F).

**Figure 7:**
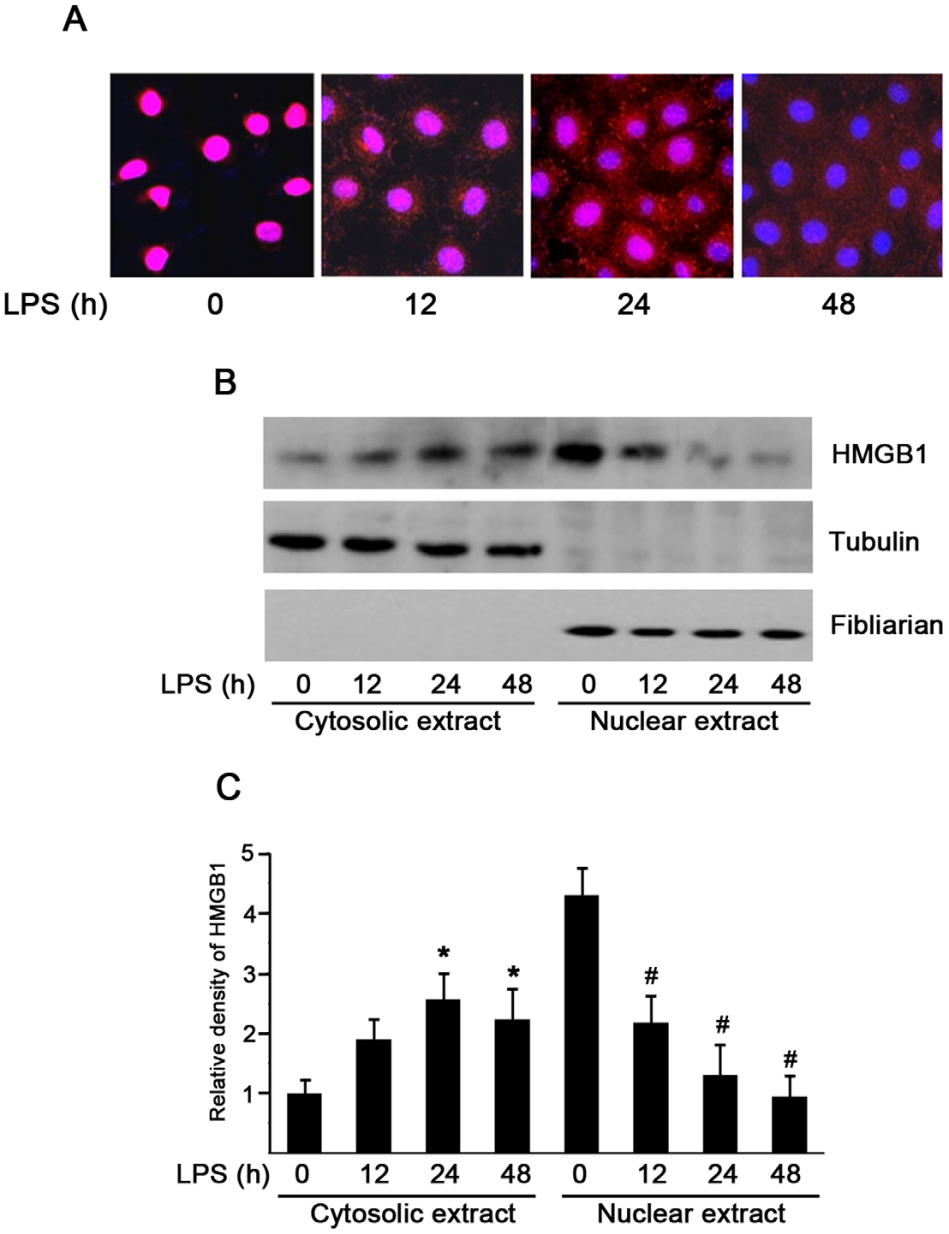
HMGB1 nuclear-cytoplasmic translocation in LPS-induced HMrSV5 cells. Cells were treated with 2 µg/ml LPS for the indicated time period. (A) Representative confocal microscopic images showed the cellular localization of HMGB1 (red) and nuclear staining (blue) by indirect immunofluorescence staining in cells. Original magnification×400. (B) HMGB1 content in cytoplasm and nuclear fractions after LPS stimulation were assessed by Western blot analysis. (C) Quantitative determination of the relative abundance of HMGB1 in the cytoplasm and the nucleus among different groups. Data are expressed as mean±SE of three experiments. *P<0.01 *versus* negative control in the cytoplasm; #*P<*0.01 *versus* negative control in the nucleus.

Because HMGB1 resides primarily in the nucleus, we further speculated the relocation of HMGB1 from the nucleus to the cytoplasm after LPS treatment. As shown by immunofluorescence, HMGB1 was noted predominantly in the nucleus in the absence of LPS stimulation, but it appeared to move from the nucleus to the cytoplasm by displaying a punctuate staining in both nucleus and cytoplasm in the presence of LPS treatment for 24 hr (Fig. 7A). To further confirm those findings, cytoplasm and nuclear fractions were isolated and subjected to immunoblot analysis with antibodies specific for HMGB1, fibrillarin (a marker of nuclear protein) and tubulin (a marker of cytoplasm protein), respectively. Consistent with immunofluorescence results, HMGB1 was located primarily in the nucleus under the baseline condition. LPS treatment resulted in HMGB1 increase in the cytoplasm and corresponding decrease in the nucleus (Fig. 7B and C). Both immunofluorescence staining and immunoblot analysis indicate that LPS treatment causes HMGB1 nuclear-cytoplasmic translocation before releasing it into the extracellular milieu in peritoneal mesothelial cells.

### HMGB1 was Secreted via Secretory Lysosome by HMrSV5 Cells

**Figure 8:**
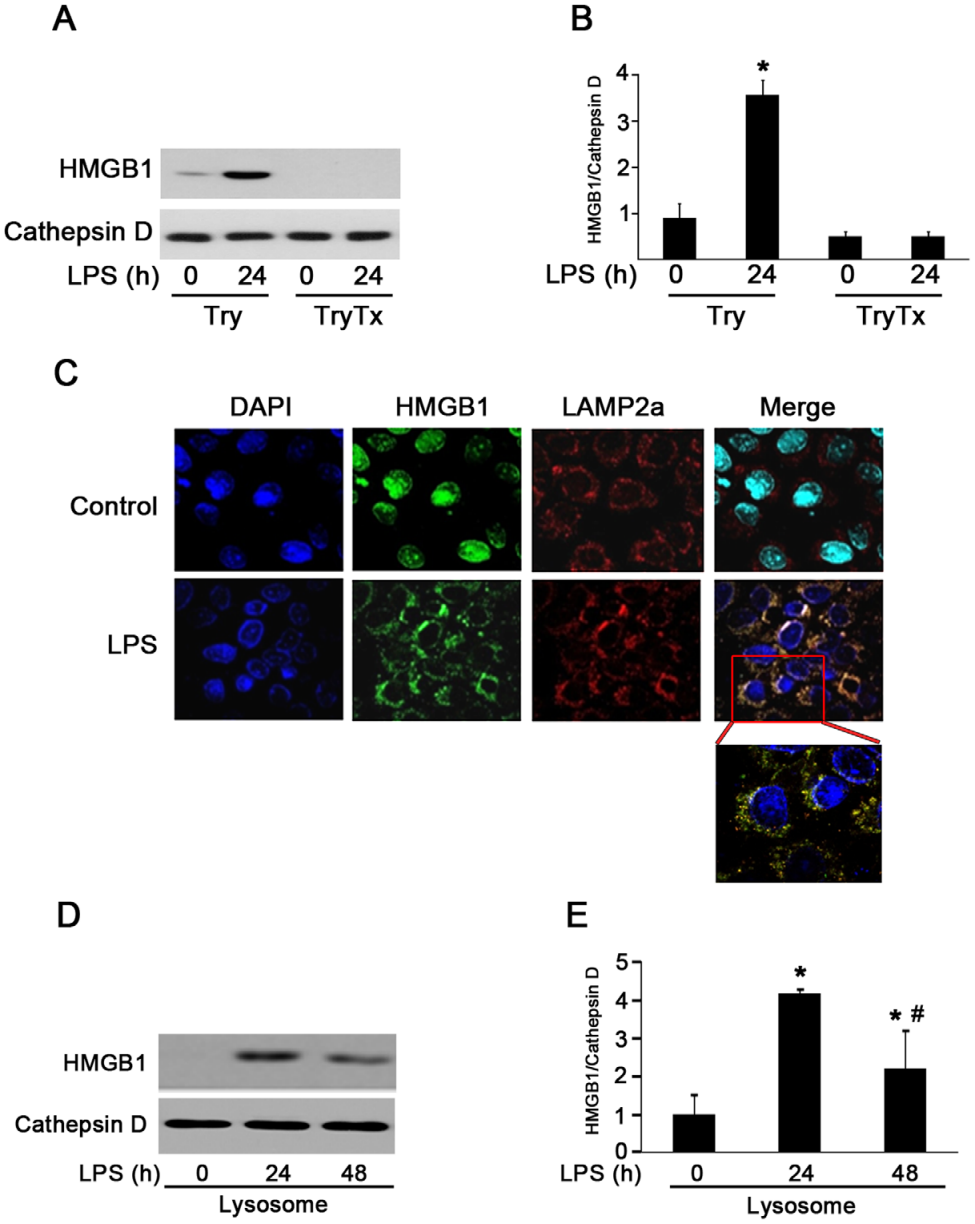
HMGB1 was secreted via lysosome-mediated secretory pathway in response to LPS stimulation. (A). HMGB1 was present in vesicles cofractionating with lysosomes after LPS administration. Lysosome fractions were untreated (lanes 1 and 2, Try) or solubilized (lanes 3 and 4, TyrTx) with Triton X-100 (TX) before trypsin (Try) digestion and subjected to Western blot analysis with anti-Cathepsin D and anti-HMGB1 antibodies. (B) Densitometry of HMGB1 content in different groups. Data are expressed as mean ± SE, *n = *3 per treatment, *P<0.05 *versus* Try treated only group. (C) Cells were treated with LPS (2 ug/ml) for 24 hr. Representative immunofluorescence analysis of cellular localization of HMGB1 (green) and LAMP2a (red), a maker of lysosome in cells. Original magnification×400. (D) HMGB1 protein contents in lysosome fractions following LPS treatment were determined by Western blotting. (E) Quantitative determination of the relative abundance of HMGB1 among different groups. Data are expressed as mean ± SE, *n = *3 per treatment, *P<0.05 *versus* control group, # *P*<0.05 *versus* LPS treated for 24 hr.

HMGB1 shuttles continually from the nucleus to the cytoplasm in quiescent cells [19]. However, HMGB1 lacks a leader peptide and thus not secreted via the Golgi/ER pathway. It has been reported that HMGB1 secretion was mediated by secretory lysosomes in innate immune cells, such as macrophages/monocytes [20]. To test whether peritoneal mesothelial cells share the same secretory pathway with macrophages, LPS-activated HMrSV5 cells were homogenized. Lysosome fractions were collected by a Lysosome Enrichment Kit and subjected to immunoblot analysis. Cathepsin D was used as a marker for the purity of lysosome fraction. As shown in Figure 8A and B, a few amount of HMGB1 was present in lysosome extracts under a baseline condition and showed a marked increase after LPS treatment (lane 1 and 2). In contrast, HMGB1 was not detectable in the presence of Triton X-100 (lane 3 and 4). The distribution of HMGB1, as revealed by the immunofluorescent staining, further confirmed an extensive colocalization of HMGB1 with the lysosome marker LAMP2a (Fig. 8C).

We showed that LPS stimulated a time-dependent release of HMGB1. To explore whether the increase of extracellular HMGB1 levels was associated with a reduction of lysosomal levels, the lysosomes were isolated and examined for HMGB1. After LPS exposure for 48 hr, lysosomal HMGB1 levels significantly decreased (Fig. 7D and E), suggesting that HMGB1 nuclear-cytoplasmic translocation induced by LPS may locate in a dense vesicular compartment of lysosome and secrete upon exocytosis of these organelles.

## Discussion

In this study, we first demonstrated a significant elevation of HMGB1 in PDE of PD patients with clinical peritonitis. Increased HMGB1 levels gradually declined during the period of effective antibiotic treatments. Moreover, HMGB1 levels were markedly and positively correlated with WBC counts and with TNF-α and IL-6 levels. Inhibition of HMGB1 augmented peritoneal inflammation and improved peritoneal function in LPS-associated peritonitis in mice. *In vitro* studies showed that LPS induced HMGB1 nuclear-cytoplasmic translocation in peritoneal mesothelial cells. Upon low doses of LPS stimulation, HMGB1 was released actively into extracellular media and cytosolic HMGB1 secreted via a lysosome-mediated secretory pathway. These findings provide significant insights that HMGB1 is involved in PD-related peritonitis process and elevated HMGB1 in PDE are actively secreted, at least in part, from activated peritoneal mesothelial cells.

HMGB1 has been implicated as a key mediator of inflammatory disease. It is an actively secreted cytokine from innate immune cells during infection. HMGB1 triggers, amplifies and extends the inflammatory response by inducing cytokine release and mediating cell injury and necrosis [16], [21], [22]. A large number of evidences have shown that pharmacologic inhibition of HMGB1 activity (antibodies, antagonist proteins, release inhibitors) in animals confers protection against various infectious inflammatory diseases, suggesting a pathogenic role and important biological activities for extracellular HMGB1 in local or systemic inflammation [16], [23]. However, it has never been investigated whether HMGB1 levels are elevated in PDE of patients with peritonitis. Our study showed that PDE HMGB1 levels in patients with peritonitis were significantly higher than those in control subjects and correspondingly elevated levels decreased gradually after effective antibiotic treatment. Analysis of a subgroup of patients revealed that patients with Gram-negative peritonitis had greater HMGB1 levels in PDE compared with Gram-positive peritonitis, which underlying cause(s) has not been fully understood. Consistent with other previous reports, our data showed that PDE levels of TNF-α and IL-6 were markedly elevated on the first day of peritonitis. Importantly, there was a significant positive correlation between PDE levels of HMGB1 and TNF-α, IL-6, as well as WBCs counts during peritonitis. In line with previous studies [11], [24], [25], [26], our results also showed that inhibition of HMGB1 by glycyrrhizin significantly attenuated LPS-induced peritoneal inflammatory cells infiltration and improved peritoneal function in mice, supporting the potential pathogenic role of HMGB1 in LPS-associated peritonitis. Taken together, these findings indicate that the HMGB1 levels in the PDE may be related to the process and severity of PD-related peritonitis.

The peritoneal macrophages in peritoneal cavity form the first line of defense against invading microorganisms. These cells are activated by microbial components resulting in complement activation and the release of proinflammatory mediators. It has been reported that both immune and non-immune cells actively release HMGB1 [5], [16], [19], [23]. There is also evidence suggesting that the peritoneal mesothelial cells play a pivotal role in the local defense by their ability to produce various cytokines [27], [28]. However, it is unclear whether HMGB1 can be expressed and released by mesothelial cells during peritonitis. Our studies showed that at nontoxic concentrations of LPS exposure, the release of HMGB1 in HMrSV5 cells was not dependent on cell death, suggesting that the active release of HMGB1 occurred in peritoneal mesothelial cells following a sublethal dose of LPS stimulation. However, at slight cytotoxic dosages, LPS (5 µg/ml) might also cause passive HMGB1 leakage from peritoneal mesothelial cells. These findings support the notion that peritoneal mesothelial cells might be a source or additional source for extracellular HMGB1.

As a non-histone chromosomal protein, HMGB1 localizes mainly in the nucleus of most cells under basal condition. It has been reported that HMGB1 secretion from monocytes/macrophages depends on relocalization from the nucleus to special cytoplasmic organelles, the secretory lysosomes [20]. In agreement with previous studies, we also observed that LPS induced HMGB1 nuclear-cytoplasmic translocation and once moving in the cytoplasm of peritoneal mesothelial cells, HMGB1 was loaded into secretory lysosomes. More importantly, these alterations were associated with the corresponding increased HMGB1 levels in extracellular milieu, supporting a lysosome-mediated HMGB1 export from LPS-stimulated human mesothelial cells.

In conclusion, our study reveals that HMGB1 levels in PDE are elevated and may play a critical role in peritoneal dysfunction during PD-related peritonitis. Activated or damaged peritoneal mesothelial cells may contribute to the increased HMGB1 in PDE. Thus, we propose that blockage of HMGB1 might represent a potential therapeutic strategy in PD-related peritonitis.

## Materials and Methods

### Ethics Statement

The study protocol was approved by the Ethics Committee of the First Affiliated Hospital, Sun Yat-sen University (Guangzhou, China). All participants provided written informed consent.

### Patients

This was a longitudinal observational study. 42 PD patients within 24 hr of the onset of the first clinical signs and symptoms of peritonitis during the period from August 2008 to May 2009 in our PD center were recruited in the study. 19 PD patients without peritonitis were randomly selected during the study period and served as controls. The two groups were comparable in age, sex, primary renal disease, duration of dialysis, and co-morbidities. Peritonitis was defined as the presence of two of the following criteria: abdominal pain, cloudy effluent with ≥100 white blood cells (WBC)/µl and ≥50% polymorphonuclear cells, or positive dialysate microbiological culture [29]. Episodes of peritonitis were initially treated with intraperitoneal ceftazidine, 1.0 g/2 L, and cefazolin, 1.0 g/2 L for one exchange daily for 14 days. The antibiotic regimen was modified on the basis of organism identification and drug sensitivity. Patients with PD-associated peritonitis were divided into two groups (Gram-positive and Gram-negative) based on the results of the Gram stain and the microbiological culture. Subjects with polymicrobial, fungal peritonitis, other organisms or culture negative were excluded.

All samples were taken when the patients were entered into this study. WBCs in PDE and serum haemoglobin were analyzed by standard techniques. Serum urea, creatinine, total cholesterol, triglycerides and albumin were measured by an autoanalyser (COBAS INTEGRA 400 plus, Roche). Serum hs-CRP was measured by using a latex enhanced immunoturbidimetric method with detection limit 0.07 mg/L (Roche Diagnostic, Mannheim, Germany). Residual GFR was defined as the average of 24-hr urinary urea and creatinine clearances. Total Kt/V was calculated using the PD Adequest 2.0 computer program for Windows (Baxter Healthcare Corp, Deerfield, IL).

### Collection of PDE

The serial PDE samples were collected before the initiation of antibiotic treatment and after 1, 3, 7 and 14 days of treatment. All PDE samples were overnight dialysate effluent (1.36 g/dL glucose concentration) and examined by routine microbiology laboratory analysis and microbiological culture. The remaining dialysate was centrifuged at 4000×g for 15 min. The supernatants were collected and stored at −80°C until analysis.

### Reagents and Antibodies

Reagents were obtained from the following sources: LPS (*Escherichia coli serotype 0111*:*B4*) and Glycyrrhizin were from Sigma-Aldrich (St. Louis, MO, USA). Anti-HMGB1 and anti-fibrillarin antibodies were purchased from Abcam (Cambridge, MA, USA). Anti-Cathepsin D was from Calbiochem (Merck, Darmstadt, Germany). Anti-β-tubulin was from Boster Biological Technology (Wuhan, China). Horseradish peroxidase (HRP) -conjugated anti-mouse IgG, HRP-conjugated anti-rabbit IgG and Alexa Fluor 546-conjugated anti-rabbit IgG were purchased from Cell Signal Technology (Beverly, MA, USA).

### Enzyme-linked Immunosorbent Assay (ELISA)

The concentrations of HMGB1 (HMGB1 ELISA Kit II, Shino-Test Corporation, Tokyo, Japan), TNF-α and IL-6 (R&D Systems, Inc., Minneapolis, MN, USA) in PDE of patients were measured by using commercially available ELISA kits according to the manufacturer’s instructions. The sensitivity of the HMGB1, TNF-α and IL-6 was 1 ng/mL, 0.70 pg/mL and 1.6 pg/mL, respectively. Each sample was run in triplicate and compared with a standard curve. The mean concentration was determined for each sample.

### Animal Studies

Adult male C57 BL/6J mice (20–25 g) were obtained from Guangdong Medical Experimental Animal Center (Guangzhou, China). The animal experimental protocols were approved by Animal Care and Use Committee of the Sun Yat-sen University. Acute peritonitis was generated in mice by intraperitoneal injection of a single dose of LPS (10 mg/kg) in 1 ml sterile saline, as previously described [14], [30]. 10 mg/mouse glycyrrhizin was administrated intraperitoneally 1 hr before LPS treatment. Glycyrrhizin was diluted in 50 mM NaOH at 37°C and pH was adjusted to pH 7.0–7.5 by the addition of 1 M Tris-HCl [15]. Control mice received the same volume sterile saline. Peritoneal function was evaluated by a 2-hr peritoneal equilibration test (PET) at 48 hr after LPS injection, as previously described [14]. The dialysate and blood samples were collected during PET. Dialysate WBCs and the differential count were counted by an automatic hematology analyzer. Concentrations of urea nitrogen and glucose in serum and dialysate were measured using an autoanalyser (COBAS INTEGRA 400 plus, Roche). Peritoneal solute transport was calculated from the dialysate concentration at 2 hr relative to its concentration in the initial infused dialysis solution (D/P creatinine) for urea nitrogen, and the dialysate-to-plasma concentration ratio (D/D_0_ glucose) at 2 hr for glucose. Peritoneal tissues were collected and subjected to immunohistochemical analysis using hematoxylin-eosin (HE) staining.

### Cell Culture and Viability

The in *vitro* studies were performed in human peritoneal mesothelial cell line (HMrSV5) as previously reported [14]. Cells were cultured in DMEM Nutrient Mix F12 media (Invitrogen Life Technologies, Carlsbad, CA, USA) supplemented with 10% fetal bovine serum. Cells were grown to approximately 70–80% confluence and subjected to serum-deprivation for 24 hr before LPS exposure. Cell viability was determined by the MTT [3-(4, 5-dimethylthiazol-2-yl)-2, 5-diphenyl tetrazolium bromide] test.

### Preparation of the Cell Culture Media and Cellular Extracts

After exposure to the indicated experimental conditions, cell media were collected and filtered through Millex-GP (Millipore, Bedford, MA) to remove cell debris and macromolecular complexes. Superntants were then concentrated with Amicon Ultra-4 -10000 NMWL (Millipore, Bedford, MA) according to the manufacturer’s instructions.

Cells were harvested and washed twice with cold PBS. Nuclear and cytoplasmic extracts were isolated as in our previous study using NE-PER Nuclear and Cytoplasmic Extraction Reagents (Pierce, Rockford, IL, USA) [31].

Lysosome-enriched fractions were enriched by using the Lysosomes Extract Kit following the manufacturer’s instruction (Pierce, Rockford, IL, USA). In brief, cells with or without LPS treatment were harvested and cell pellets were incubated with Lysosome Enrichment Reagent A and B, then centrifuged at 500×g for 10 min at 4°C to remove nuclei and cell debris. Post-nuclear supernatants were mixed with the OptiPrep™ Cell Separation Media (Pierce, Rockford, IL, USA) to make a final concentration of 15% OptiPrep™ Media and subjected to gradient centrifugation. Lysosome lysis was collected from the top of the gradient, treated with 100 µg/ml trypsin for 30 min on ice, in the absence or presence of 1% Triton X-100, and subjected to immunoblot analysis.

### Western Blot Analysis

Proteins were isolated from PDE samples, cell culture media or cell extracts. The protein concentration was measured by the Bradford protein assay (Bio-Rad, Hercules, CA). Equal volume (40 µl) of the concentrated PDE sample or cell culture media and equal amounts of proteins from cellular extracts were loaded and separated by 10% SDS-PAGE and transferred onto nitrocellulose membrane. The blots were probed overnight at 4°C with specific antibodies, and detected using the ECL system as previously described [31]. Densitometric analysis was performed with the image analysis program (FluorChem 8900; Alpha Innotech Corp, San Leandro, CA).

### Immunoﬂuorescence and Immunohistochemical Analysis

Indirect immunofluorescence staining was performed following conventional procedures as described in our previous study [31]. Briefly, cells were cultured on glass coverslips and stimulated with 2 µg/ml LPS for the indicated time. Subsequently, cells were fixed in 4% formaldehyde, permeabilized with 0.1% Triton-X-100 in PBS and sequentially incubated with the indicated primary antibodies, washed 3 times in PBS and incubated for 1 hr with each of the corresponding secondary antibodies. All images were collected by a laser scanning confocal microscopy (Zeiss LSM 510 META, Carl Zeiss, Germany).

Both parietal and visceral peritoneum were fixed in 10% phosphate buffer formalin, dehydrated through graded alcohol and xylene, embedded in paraffin, sectioned at 2-µm thickness, and then stained with hematoxylin and eosin. Histological examinations were observed by light microscopy and evaluated in a blinded manner.

### Statistical Analysis

Continuous data were expressed as mean ± standard deviation (SD) or median (interquartile range IQR) and categorical data were expressed as frequencies and percentage. Differences among groups were analyzed by one way-ANOVA analysis. Significant variation in the data within groups was investigated using a Kruskal Wallis test. Frequency data (e.g., gender) were analyzed by the Chi-square test. The Spearman or Pearson correlation coefficient test was used to evaluate associations between two quantitative variables. *P* values <0.05 were considered significant. All statistical analyses were conducted using SPSS 15.0 statistics software (version 15.0, SPSS Inc, Chicago, IL).
